# A novel approach for the treatment of Jacob II and III fractures of the lateral humeral condyle in children: Percutaneous Kirschner wire fixation with ultrasound localization

**DOI:** 10.3389/fsurg.2022.1000399

**Published:** 2022-11-07

**Authors:** Changzong Deng, Zhien Shen, Kai Wang, Wenbin Xu, Weibin Du, Wei Zhuang

**Affiliations:** ^1^ Research Institute of Orthopedics, The Affiliated Jiangnan Hospital of Zhejiang Chinese Medical University, Hangzhou, China; ^2^ Hangzhou Xiaoshan Hospital of Traditional Chinese Medicine, Hangzhou, China

**Keywords:** ultrasound, children, lateral humeral condyle fractures, minimally invasive, treatment

## Abstract

This research investigated the effectiveness of percutaneous Kirschner wire fixation in children with Jacob II and III lateral humeral condyle fractures. 28 children with Jacob II and III lateral humeral condyle fractures were treated with percutaneous Kirschner wire fixation under ultrasound localization, followed by cast immobilization for 4–5 weeks at our institution from January 2018 to April 2022. X-rays were evaluated on the first postoperative day to assess fracture reduction and Kirschner wire fixation. After 2 and 4 weeks, x-rays were taken to assess fracture healing and the presence of discomfort and infection was evaluated. After confirming fracture healing and callus formation, the cast and Kirschner wire were removed. Rehabilitation exercises were conducted following removal to restore elbow function. At the last follow-up, most results were excellent (*n* = 25) and good (*n* = 3) according to Flynn's criteria. Moreover, according to the Mayo Elbow Functional Score Scale (MEPS), all 28 children had excellent scores, with no significant difference in MEPS scores between the lesion and healthy sides (*t* = 1.533, *p* > 0.05). The present study substantiated that our novel approach is more convenient and effective, brings less trauma and complications and no radiation and deserves clinical promotion.

## Background

It is well-established that fractures of the lateral condyle of the humerus are common elbow fractures in children, second only to supracondylar fractures, occurring mostly between ages 5 and 10 and are responsible for 15 to 20% of elbow fractures in this patient population (1, 2). The mechanism of injury generally involves forearm varus or valgus stress exerted by the elbow in extension. Heavy, obese children are at greater risk for lateral humeral condyle fractures (3). Jacob's classification is clinically used to classify lateral humeral condyle fractures according to the fracture displacement: type I: articular surface continuity; type II: articular surface fracture; type III: fracture fragment rotation. Jacob type I fractures are treated conservatively with cast immobilization, while Jacob type III fractures generally require open reduction with Kirschner wires, screws, or cannulated nails due to considerable displacement. However, much controversy surrounds the optimal treatment for Jacob type II fractures, with inconsistent reports on open or closed reduction treatment.

Manual reduction with percutaneous Kirschner wire fixation under arthrography is the mainstay of treatment. However, arthrography requires repeated x-ray fluoroscopy, which increases the risk of iatrogenic radiation damage and is an invasive procedure with hazards such as allergies to contrast agents. In recent years, ultrasound has been widely employed in treating lateral condyle fractures in children due to its ability to visualize cartilage hinges, properly determine re-displacement risk, and avoid iatrogenic harm (4, 5). Therefore, the clinical efficacy of percutaneous Kirschner wire fixation for children with Jacob II and III fractures of the lateral humeral condyle under ultrasound localization was assessed in the present study.

## Patients and data

A total of 28 cases of Jacob II (*n* = 21) and III (*n* = 7) fractures of the lateral humeral condyle were included, with a mean age of 5.5 ± 2.1 years (range 2–10 years) and consisting predominantly of males (*n* = 20). The mean time from injury to surgery was 2.6 ± 0.8 days (range 1–4 days). The same surgical team completed all surgical operations in this study, and the legal guardians of the children agreed to participate and signed the informed consent form. A Hitachi F31 ultrasound instrument with an 8 MHz high-frequency probe (Hitachi, Japan) was used in this study (Table 1).

**Table 1:** Patients and data.

Case	Age	Sex	Jacob's type	K-wires number	K-wires Size (mm)	Time from injury to surgery (day)	operation time (min)	Plaster removal time (day)	limited flexion	carrying angle	Evaluation
1	7	1	2	2	1.6	3	30	35	3°	3°	Excellent
2	8	1	2	2	1.6	4	30	35	2°	3°	Excellent
3	8	1	2	2	1.6	1	32	30	0°	0°	Excellent
4	8	1	2	2	1.6	2	30	32	3°	2°	Excellent
5	6	2	2	2	1.6	3	33	30	0°	0°	Excellent
6	2	2	2	2	1.4	4	30	32	3°	2°	Excellent
7	6	1	2	2	1.6	3	30	32	0°	0°	Excellent
8	7	2	2	2	1.6	3	30	35	5°	3°	Excellent
9	3	1	2	2	1.4	3	30	34	0°	0°	Excellent
10	4	2	2	2	1.6	2	35	35	3°	3°	Excellent
11	6	2	2	2	1.6	2	35	35	0°	0°	Excellent
12	2	1	2	2	1.6	2	30	35	5°	5°	Excellent
13	6	1	2	3	1.6	4	30	35	0°	0°	Excellent
14	10	1	2	3	1.6	3	35	35	2°	2°	Excellent
15	4	2	2	3	1.6	2	30	35	2°	2°	Excellent
16	4	1	2	2	1.4	2	30	34	0°	0°	Excellent
17	4	1	2	2	1.6	2	34	35	3°	5°	Excellent
18	6	1	2	2	1.4	2	30	35	0°	0°	Excellent
19	4	1	2	3	1.6	1	30	35	2°	2°	Excellent
20	8	1	2	3	1.8	4	30	35	3°	3°	Excellent
21	5	1	2	3	1.6	2	35	35	4°	5°	Excellent
22	5	1	3	3	1.6	3	35	35	9°	9°	Good
23	6	1	3	3	1.6	3	38	35	5°	4°	Excellent
24	3	2	3	3	1.6	3	35	35	8°	6°	Good
25	10	2	3	2	1.6	3	35	35	4°	3°	Excellent
26	5	1	3	3	1.6	3	35	35	5°	3°	Excellent
27	3	1	3	3	1.6	2	35	34	3°	4°	Excellent
28	6	1	3	3	1.6	2	35	35	9°	6°	Good

Sex: 1: male, 2: female.

This retrospective study was approved for publication by the ethics committee of Jiangnan Hospital Affiliated with the Zhejiang University of Traditional Chinese Medicine (Hangzhou Xiaoshan Hospital of Traditional Chinese Medicine). (XSZYY2081115) and conducted based on the tenets of the Declaration of Helsinki. Copies of the written consent form are available for review by the editors of this journal. The study is reported in agreement with the principles of the CAse REport (CARE) guidelines.

## Inclusion and exclusion criteria

### Inclusion criteria

1. Age ≤ 10 years old; 2. x-ray imaging (including frontal and lateral films) showing Jacob II and III fractures of the lateral condyle of the humerus; 3. Absence of vascular and nerve damage at the time of injury; 4. Follow-up duration >6 months.

### Exclusion criteria

1. Age > 10 years old; 2. x-ray imaging (including frontal and lateral films) showing Jacob type I fracture of the lateral condyle of the humerus; 3. Patients with concomitant elbow injuries, including supracondylar and intercondylar fractures of the humerus, olecranon fractures, and radial head and neck fractures. 4. Presence of neurovascular complications; 5. Refusal of surgery; 6. Loss to follow-up.

## Treatment methods

### Preoperative treatment

After admission, the child received symptomatic treatment for analgesia and detumescence. All children underwent preoperative examinations to assess the surgical risk.

### Surgical methods

**Figure 1:**
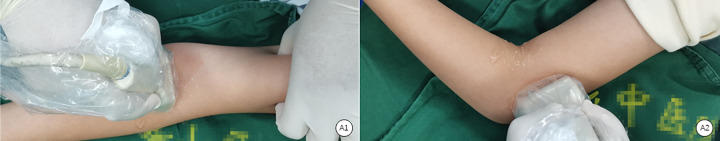
(**A1**) The transverse plane. (**A2**) The coronal plane.

**Figure 2:**
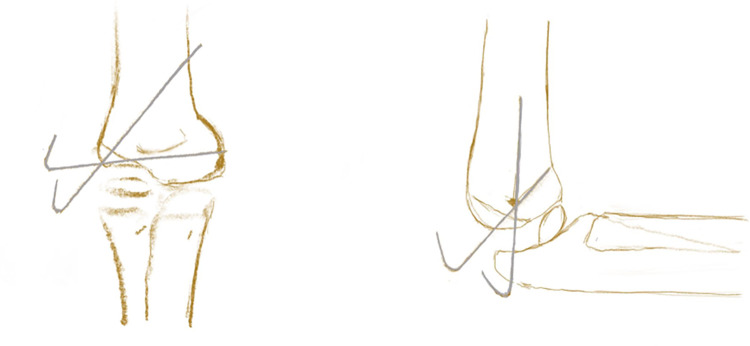
Intraoperative K-wire insertion angle.

**Figure 3:**
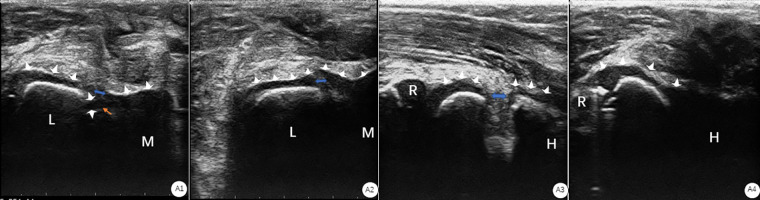
(**A1**) Ultrasonography on the transverse plane showed that the cartilage hinge (white arrow) was broken (blue arrow), the “step sign” was seen at the broken end, and the triangular bone fragment (yellow arrow) at the broken end was free from the cartilage articular surface. (Note: L the humerus Lateral condyle; M: the medial condyle of the humerus). (**A2**) Ultrasonography on the transverse plane showed that the cartilage hinge (white arrow) was broken (blue arrow), and the “step sign” was seen at the broken end. (**A3**) Ultrasonography on the coronal plane showed that the cartilage hinge (white arrow) was broken (blue arrow), and the “step sign” was seen at the broken end. (Note: **R**: radial head; **H:** humeral shaft). (**A4**) Ultrasonography on the coronal plane shows the continuity of the cartilage hinge (white arrow). (Note: it radial head; humeral shaft).

Operation process: After a combination of brachial plexus block with general anesthesia, the surgical site was draped. Hematomas in the joint cavity were aspirated with a needle when present. Preoperative sterile probe preparation, apply sterile medical ultrasonic couplant on the probe surface and wrap it with sterile membrane (Figure 1). During intraoperative ultrasound, the probe was predominantly placed along the transverse and coronal plane. When the elbow was in a flexed position, the ultrasound probe was used to visualize the lateral elbow in the coronal plane and assess the condition of the cartilage at the distal humerus (Figure 1). Total separation, articular surface displacement, or lateral condyle rotation could be observed. Sufficient traction should be given before reduction and rotated type III fracture pieces should be reduced by gentle manipulation. Under ultrasound guidance, the rotationally displaced fracture fragments were reduced, and the reduced type III fracture was transformed into a type II fracture. Simultaneously, valgus pressure was applied, the elbow joint was flexed and stretched to align the articular surfaces, and the “steps” on ultrasound disappeared, indicating effective articular surface reduction (Figure 2). Under ultrasound guidance, 2–3 Kirschner wires (size 1.50–1.80 mm) were inserted parallel from the lateral condyle and the proximal end of the fracture at an angle of 45° to the articular surface, with extra caution to avoid nerves and blood vessels (Figure 3). The fractured end was reduced under x-ray fluoroscopy, and elbow flexion and extension were excellent. After the operation, the needle port was cleaned, and the Kirschner wire was bent and cut for outpatient removal. The average intraoperative blood loss was about 10.00 ± 5.00 ml.

### Postoperative management

X-rays were taken on the first day after surgery to assess fracture reduction and Kirschner wire fixation. After 2 and 4 weeks, x-rays were repeated to assess fracture healing and patients were assessed for any discomfort or signs of infection. The cast and Kirschner wires were removed after a mean duration of 34.21 ± 1.52 days after confirming fracture healing and callus formation. After removal, the patients underwent rehabilitation exercises to improve recovery of elbow function.

## Results

### Efficacy evaluation indicators

At 2, 4 weeks, 3, and 6 months after surgery, anterior and lateral elbow x-rays were collected to assess fracture healing. The Flynn and Mayo Elbow Score (MEPS) was used to evaluate the children's postoperative functional recovery.

Flynn's criteria (6) is commonly used to evaluate outcomes into four grades based on the carrying angle and motion loss. Excellent, Good, Fair, and Poor results were associated with a 0–5°, 6–10°; Fair, 11–15° and over 15° motion loss and carrying angle.

The Mayo Elbow Joint Function Rating Scale (7) scores the function of the affected limb based on pain, range of motion, stability, and daily functional activities, with a full score of 100. Excellent, good, moderate and poor outcomes were associated with scores of over 90, 75–89, 60–74, and below 60, respectively.

## Treatment results

### Elbow joint function

**Figure 4:**
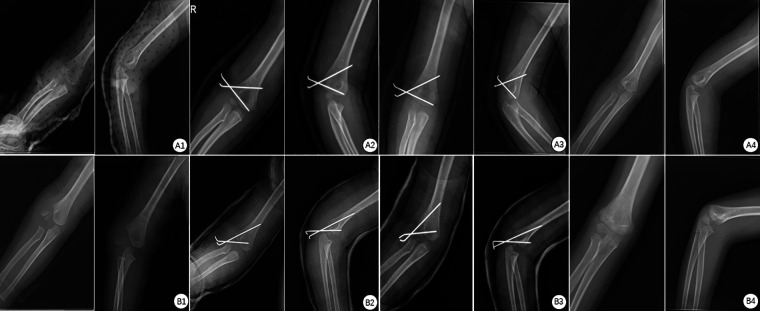
(**A1**) Pre-operative type II frontal and lateral view: fracture of the articular surface, displacement <2mm. (**A2**) Frontal and lateral view on the first day after operation of type II: the Kirschner wire was stable and the articular surface was continuous. (**A3**) Frontal and lateral view of type II one month after operation: the Kirschner wire was stable and the callus formed at the fracture end. (**A4**) Frontal and lateral view two weeks after the plaster removal of type II after operation: the fracture healed well.B1. Pre-operative type III frontal and lateral view: fracture of the articular surface, rotational displacement >2 mm. (**B2**) Frontal and lateral view on the first day after operation of type III: the Kirschner wire was stable and the articular surface was continuous. (**B3**) Frontal and lateral view of type III in the second week after operation: the Kirschner wire was stable and the callus formed at the fracture end. (**B4**) Frontal and lateral view twelve months after the removal of the plaster of type III after operation: the fracture healed well.

All 28 children were observed for 6–12 months (mean 8.40 ± 2.30 months). The average operation duration was 32.39 ± 2.60 min. No infection, fracture displacement, delayed union, fishtail deformity, early epiphyseal closure, growth arrest, nonunion, functional impairment, or postoperative arthritis complications were detected. All fractures healed after Kirschner wire removal. The average recovery time was 5.40 ± 0.50 weeks (Figure 4).

At the last follow-up, according to Flynn's criteria, results were excellent in 25 cases and fair in 3 cases. No children presented with a loss of carrying angle of 11–15° or >15° (Table 1).

At the final follow-up, all 28 children had excellent MEPS scores. There was no significant difference in MEPS (99.80 ± 0.39) between the lesion side and the healthy side (99.92 ± 0.17) (*t* value = 1.533, *p* value > 0.05) (Table 2).

**Table 2:** Percutaneous Kirschner wire fixation of children with Jacob II and III fractures of the lateral humeral condyle under ultrasound localization in 28 children with MEPS comparison at the last follow-up.

Side	Pain	AROM	Stability	ADL	Score
lesion	45.00 ± 0	19.96 ± 0.18	9.92 ± 0.26	24.91 ± 0.27	99.80 ± 0.39
healthy	45.00 ± 0	19.98 ± 0.09	9.96 ± 0.13	25.00 + 0	99.92 ± 0.17
T value		0.447	0.645	1.724	1.533
*p* value		0.657	0.522	0.096	0.134

AROM, active range of motion; ADL, activities of daily living.

## Discussion

The benefits and drawbacks of ultrasound localization in the treatment of lateral humeral condyle fractures in children.

It is well-established that ultrasound localization is better than arthrography for treating lateral humeral condyle fractures in children (8) for the following reasons: 1. The absence of ionizing radiation minimizes radiation damage from frequent x-ray fluoroscopy. 2. This approach is more convenient and fracture fragments can be seen from multiple directions and joint planes. 3. Ultrasound guidance provides greater precision, avoiding unnecessary surgery for children with mildly displaced lateral humeral fractures. 4. Nerve and blood vessel damage can be seen before or during the operation. 5. Epiphysis and cartilage injury can be prevented. 6. Intraoperative dynamic monitoring of the cartilage hinge. 7. Intraoperative trauma is minimal, with relatively lower bleeding and reduced iatrogenic harm. The short surgery duration promotes early fracture repair and functional recovery of the elbow joint.

Although percutaneous Kirschner wire repair of lateral humeral condyle fractures in children under ultrasound localization has numerous benefits, many drawbacks have been reported (9). For instance, ultrasonography requires experienced operators with professional training. Accordingly, the high learning costs may hinder the wide implementation of this approach. Moreover, it should be borne in mind that ultrasound cannot be used to identify fracture displacement during plaster immobilization, unlike an x-ray.

### Treatment experience and study limitations

The lateral condyle fracture of the humerus is the second most frequent elbow fracture in children. Ultrasound technology has become extremely popular clinically, given its convenience, non-invasiveness, and ability to visualize neurovascular and soft tissues (4). Kirschner wire fixation with ultrasound localization represents a more effective approach consistent with the contemporary concept of minimally invasive surgery. During closed reduction, emphasis should be placed on the following points: 1. Gentle manipulation is essential to avoid epiphyseal injury in children leading to exacerbation of trauma and unintentional healing. 2. The Kirschner wire should be fixed at the correct angle to meet biomechanical criteria and accelerate fracture healing. 3. The tip of the needle should reach the contralateral bone and an adequate should be left outside the skin to facilitate removal.

In this research, 28 children with Jacob II and III fractures of the lateral humeral condyle were treated with percutaneous Kirschner wire fixation under ultrasonography. All fractures exhibited fast healing after surgery. At follow-up, there was no infection, deformity, or other complications. In children with type III injuries, elbow joint function scores were slightly worse than type II. For type III fractures with a displacement of >4 mm, the degree of trauma and soft tissue injury is considerable, and the chance of postoperative joint adhesion is higher than in type II children. Following long-term follow-up and correction, some children with type III trauma exhibited poorer postoperative functional recovery than type II trauma. Notwithstanding that the efficacy of type III trauma is inferior to type II trauma, excellent results have been reported in recent years with the use of ultrasound. Based on our experience, cases with displacement >4 mm and type III trauma can be more objectively visualized. Indeed, more research is warranted for the application of ultrasound in cases with minimal displacement or type II fractures.

In conclusion, percutaneous Kirschner wire fixation in children with Jacob II and III lateral humeral condyle fractures under ultrasound localization is more convenient and effective, brings less trauma and complications and no radiation and deserves clinical promotion.

## Data Availability

The original contributions presented in the study are included in the article/Supplementary Material, further inquiries can be directed to the corresponding author/s.
